# 2-(4-Ethoxy­benzyl­idene)butanoic acid

**DOI:** 10.1107/S160053680802103X

**Published:** 2008-07-12

**Authors:** Niaz Muhammad, M. Nawaz Tahir, Zia-ur-Rehman, Saqib Ali

**Affiliations:** a Department of Chemistry, Quaid-i-Azam University, Islamabad 45320, Pakistan; b Department of Physics, University of Sargodha, Sagrodha, Pakistan

## Abstract

In the crystal structure of the title compound, C_13_H_16_O_3_, dimers are formed due to inter­molecular O—H⋯O hydrogen bonding. There exists an intra­molecular C—H⋯O hydrogen bond which forms a five-membered ring. There is also present a C—H⋯π inter­action between a methyl CH group of an ethyl group and the centroid of the aromatic ring.

## Related literature

For related literature, see: Bernstein *et al.* (1995 ▶); Burt (2004 ▶); Muhammad *et al.* (2007 ▶, 2008*a* ▶,*b* ▶,*c* ▶); Niaz *et al.* (2008 ▶).
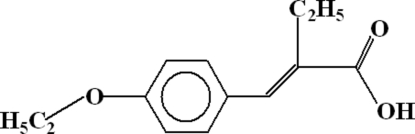


## Experimental

### 

#### Crystal data

C_13_H_16_O_3_
*M* *_r_* = 220.26
Monoclinic, 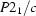

*a* = 10.3192 (7) Å
*b* = 22.0761 (15) Å
*c* = 5.2362 (3) Å
β = 100.751 (4)°
*V* = 1171.91 (13) Å^3^
*Z* = 4
Mo *K*α radiation
μ = 0.09 mm^−1^
*T* = 296 (2) K
0.26 × 0.20 × 0.16 mm

#### Data collection

Bruker Kappa APEXII CCD diffractometer
Absorption correction: multi-scan (*SADABS*; Bruker, 2005 ▶) *T*_min_ = 0.975, *T*_max_ = 0.985
13990 measured reflections
3009 independent reflections
1599 reflections with *I* > 2σ(*I*)
*R*_int_ = 0.049

#### Refinement

*R*[*F* ^2^ > 2σ(*F* ^2^)] = 0.050
*wR*(*F* ^2^) = 0.122
*S* = 1.00
3009 reflections
150 parameters
H atoms treated by a mixture of independent and constrained refinement
Δρ_max_ = 0.14 e Å^−3^
Δρ_min_ = −0.21 e Å^−3^

### 

Data collection: *APEX2* (Bruker, 2007 ▶); cell refinement: *APEX2*; data reduction: *SAINT* (Bruker, 2007 ▶); program(s) used to solve structure: *SHELXS97* (Sheldrick, 2008 ▶); program(s) used to refine structure: *SHELXL97* (Sheldrick, 2008 ▶); molecular graphics: *ORTEP-3 for Windows* (Farrugia, 1997 ▶) and *PLATON* (Spek, 2003 ▶); software used to prepare material for publication: *WinGX* (Farrugia, 1999 ▶) and *PLATON*.

## Supplementary Material

Crystal structure: contains datablocks global, I. DOI: 10.1107/S160053680802103X/at2588sup1.cif

Structure factors: contains datablocks I. DOI: 10.1107/S160053680802103X/at2588Isup2.hkl

Additional supplementary materials:  crystallographic information; 3D view; checkCIF report

## Figures and Tables

**Table 1:** Hydrogen-bond geometry (Å, °)

*D*—H⋯*A*	*D*—H	H⋯*A*	*D*⋯*A*	*D*—H⋯*A*
O1—H1⋯O2^i^	1.01 (2)	1.61 (2)	2.6184 (15)	176.8 (15)
C7—H7⋯O1	0.93	2.30	2.7218 (19)	107
C12—H12*B*⋯*CgA*^ii^	0.96	2.82	3.6534 (19)	145

Symmetry codes: (i) 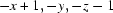; (ii) 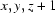. *CgA* is the centroid of the C1–C6 ring.

**Fig. 1.**
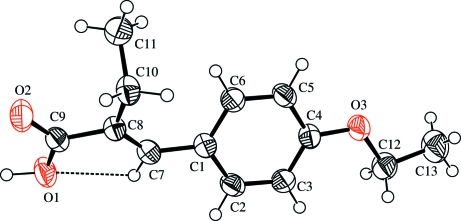
ORTEP drawing of the title compound, with the atom numbering scheme. The thermal ellipsoids are drawn at the 50% probability level. H-atoms are shown by small circles of arbitrary radii. The intramolecular H-bonds are shown by doted lines.

**Fig. 2.**
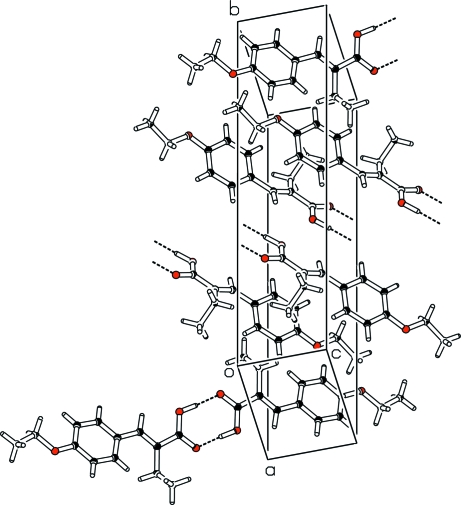
The packing figure (PLATON: Spek, 2003) which shows the dimeric nature of the compound and the interlinkages of the dimers.

## supplementary crystallographic information

### Comment

Cinnamic acid derivatives play an important role in the production of lignins for higher plants. They are well documented for their antibacterial, antifungal and antiparasitic activities (Burt, 2004). We have recently reported the various derivatives of cinamic acids (Niaz *et al.*, 2008, Muhammad *et al.*, 2008*a*) and tin complexes (Muhammad *et al.*, 2008*b*, 2008*c*). In continuation of our project, we herein report the structure of the title compound (I).

The crystal structure of 3-(4-bromophenyl)-2-ethylacrylic acid (II) (Muhammad *et al.*, 2007) differs from (I) due to the attachement of ethoxy group at *para* position instead of Br-atom. Similarly, (E) -2-(2-fluorobenzylidene) butanoic acid (III) (Niaz *et al.*, 2008) differs as the attachment of F-atom is on *meta* position. Thus, (I) is very different from the reported compounds (II) and (III).

In the crystal structure of the title compound (I), the C—C bonds are in the range 1.464 (2) to 1.511 (2) Å, and C═C have a value of 1.337 (2) Å. The value of C—O bond in the carboxylate group is 1.287 (2) Å, whereas C═O is of 1.248 (2) Å. The attached ethoxy group have 1.3595 (19) and 1.431 (2) Å as the C—O bond distances. In the asymmetric unit, there is an interamolecular H-bond of C—H···O type (Table 1, Fig 1) which completes a five membered ring (O1/C9/C8/C7/H7). Centrosymmetric dimers, *R*_2_^2^(8) (Bernstein *et al.* 1995) are formed due to the intermolecular hydrogen bonding, O1—H1···O2^i^ [symmetry code: (i) = -*x* + 1, -*y*, -*z* - 1]. These dimers have a π-interaction C12—H12B···CgA^ii^ [symmetry code: (ii) = *x*, *y*, *z* + 1] where CgA is the centroid of the aromatic ring A(C1—C6). The dihedral angle between (O1/C9/O2) and (C8/C10/C11) is 75.55 (15)°, whereas it is 30.19 (17)° between (C7/C8/C9) and (O3/C12/C13). The ethoxy group makes a dihedral angle of 2.77 (11)° with the aromatic ring. The formation of centrosymmetric dimers and packing of (I) is shown in Fig 2.

### Experimental

Compound (I) was prepared according to the reported procedure (Muhammad *et al.*, 2007). A mixture of 4-ethoxybenzaldehyde(1.39 ml, 10 mmol), ethylmalonic acid (2.64 g, 20 mmol) and piperidine (1.98 ml, 20 mmol) in a pyridine (12.5 ml) solution was heated on a steam-bath for 24 h. The reaction mixture was cooled and added to a mixture of 25 ml of concentrated HCl and 50 g of ice. The precipitate formed in the acidified mixture was filtered off and washed with ice-cold water. The product was recrystallized from ethanol [yield: 66%].

### Refinement

The H-atom of carboxylate group is taken from fourier difference map and coordinates were refined. The other H atoms were positioned geometrically, with C—H = 0.93, 0.97, and 0.96 Å for aromatic, methylene and methyl H, respectively, and constrained to ride on their parent atoms, with *U*_iso_(H) = *xU*_eq_(C, O), where *x* = 1.5 for methyl H, and *x* = 1.2 for all other H atoms.

### Crystal data

**Table:**

C_13_H_16_O_3_	*F*_000_ = 472
*M**_r_* = 220.26	*D*_x_ = 1.248 Mg m^−^^3^
Monoclinic, *P*2_1_/*c*	Mo *K*α radiation λ = 0.71073 Å
Hall symbol: -P 2ybc	Cell parameters from 3009 reflections
*a* = 10.3192 (7) Å	θ = 2.0–28.7º
*b* = 22.0761 (15) Å	µ = 0.09 mm^−^^1^
*c* = 5.2362 (3) Å	*T* = 296 (2) K
β = 100.751 (4)º	Prismatic, colourless
*V* = 1171.91 (13) Å^3^	0.26 × 0.20 × 0.16 mm
*Z* = 4	

### Data collection

**Table:**

Bruker KappaAPEXII CCD diffractometer	3009 independent reflections
Radiation source: fine-focus sealed tube	1599 reflections with *I* > 2σ(*I*)
Monochromator: graphite	*R*_int_ = 0.049
Detector resolution: 7.4 pixels mm^-1^	θ_max_ = 28.7º
*T* = 296(2) K	θ_min_ = 2.0º
ω scans	*h* = −13→13
Absorption correction: multi-scan(SADABS; Bruker, 2005)	*k* = −29→29
*T*_min_ = 0.975, *T*_max_ = 0.985	*l* = −7→6
13990 measured reflections	

### Refinement

**Table:**

Refinement on *F*^2^	Secondary atom site location: difference Fourier map
Least-squares matrix: full	Hydrogen site location: inferred from neighbouring sites
*R*[*F*^2^ > 2σ(*F*^2^)] = 0.050	H atoms treated by a mixture of independent and constrained refinement
*wR*(*F*^2^) = 0.122	*w* = 1/[σ^2^(*F*_o_^2^) + (0.0465*P*)^2^ + 0.113*P*] where *P* = (*F*_o_^2^ + 2*F*_c_^2^)/3
*S* = 1.01	(Δ/σ)_max_ < 0.001
3009 reflections	Δρ_max_ = 0.15 e Å^−^^3^
150 parameters	Δρ_min_ = −0.21 e Å^−^^3^
Primary atom site location: structure-invariant direct methods	Extinction coefficient: ?

### Special details

**Table:**

Geometry. Bond distances, angles *etc*. have been calculated using the rounded fractional coordinates. All su's are estimated from the variances of the (full) variance-covariance matrix. The cell e.s.d.'s are taken into account in the estimation of distances, angles and torsion angles
Refinement. Refinement of *F*^2^ against ALL reflections. The weighted *R*-factor *wR* and goodness of fit *S* are based on *F*^2^, conventional *R*-factors *R* are based on *F*, with *F* set to zero for negative *F*^2^. The threshold expression of *F*^2^ > σ(*F*^2^) is used only for calculating *R*-factors(gt) *etc*. and is not relevant to the choice of reflections for refinement. *R*-factors based on *F*^2^ are statistically about twice as large as those based on *F*, and *R*- factors based on ALL data will be even larger.

### Fractional atomic coordinates and isotropic or equivalent isotropic displacement parameters (Å^2^)

**Table:**

	*x*	*y*	*z*	*U*_iso_*/*U*_eq_	
O1	0.62469 (12)	−0.00644 (5)	−0.2194 (2)	0.0545 (4)	
O2	0.47639 (13)	0.06481 (5)	−0.3546 (2)	0.0577 (5)	
O3	1.08170 (11)	0.17222 (5)	1.0247 (2)	0.0506 (4)	
C1	0.82240 (15)	0.09272 (7)	0.4115 (3)	0.0388 (5)	
C2	0.89602 (16)	0.05704 (7)	0.6035 (3)	0.0437 (6)	
C3	0.98214 (17)	0.08106 (7)	0.8122 (3)	0.0447 (6)	
C4	0.99847 (16)	0.14314 (7)	0.8316 (3)	0.0396 (5)	
C5	0.92792 (17)	0.17994 (7)	0.6416 (3)	0.0457 (6)	
C6	0.84164 (17)	0.15537 (7)	0.4371 (3)	0.0461 (6)	
C7	0.73819 (16)	0.06338 (7)	0.1901 (3)	0.0420 (6)	
C8	0.63076 (16)	0.08379 (7)	0.0301 (3)	0.0385 (5)	
C9	0.57279 (17)	0.04572 (7)	−0.1946 (3)	0.0411 (5)	
C10	0.55968 (18)	0.14224 (7)	0.0567 (3)	0.0456 (6)	
C11	0.5866 (2)	0.19159 (8)	−0.1265 (3)	0.0613 (7)	
C12	1.16254 (18)	0.13667 (8)	1.2209 (3)	0.0523 (6)	
C13	1.2471 (2)	0.17960 (10)	1.4013 (4)	0.0696 (8)	
H1	0.5832 (17)	−0.0279 (8)	−0.385 (4)	0.0654*	
H2	0.88696	0.01518	0.59100	0.0525*	
H3	1.02879	0.05571	0.93877	0.0537*	
H5	0.93908	0.22172	0.65263	0.0548*	
H6	0.79466	0.18098	0.31205	0.0553*	
H7	0.76348	0.02428	0.15510	0.0503*	
H10A	0.58522	0.15670	0.23393	0.0547*	
H10B	0.46558	0.13434	0.02599	0.0547*	
H11A	0.56467	0.17725	−0.30240	0.0919*	
H11B	0.67825	0.20238	−0.08728	0.0919*	
H11C	0.53385	0.22647	−0.10632	0.0919*	
H12A	1.21722	0.10923	1.14239	0.0627*	
H12B	1.10791	0.11292	1.31526	0.0627*	
H13A	1.30278	0.20185	1.30692	0.1044*	
H13B	1.30068	0.15718	1.53886	0.1044*	
H13C	1.19191	0.20730	1.47359	0.1044*	

### Atomic displacement parameters (Å^2^)

**Table:**

	*U*^11^	*U*^22^	*U*^33^	*U*^12^	*U*^13^	*U*^23^
O1	0.0617 (9)	0.0404 (7)	0.0544 (7)	0.0025 (6)	−0.0076 (6)	−0.0162 (5)
O2	0.0623 (9)	0.0493 (8)	0.0515 (7)	0.0067 (6)	−0.0149 (7)	−0.0126 (6)
O3	0.0561 (8)	0.0421 (7)	0.0456 (7)	−0.0019 (6)	−0.0109 (6)	−0.0018 (5)
C1	0.0377 (10)	0.0378 (9)	0.0402 (9)	−0.0003 (7)	0.0053 (8)	−0.0036 (7)
C2	0.0484 (11)	0.0316 (9)	0.0501 (10)	−0.0018 (8)	0.0066 (9)	−0.0015 (7)
C3	0.0477 (11)	0.0397 (10)	0.0434 (10)	0.0012 (8)	−0.0002 (8)	0.0037 (7)
C4	0.0391 (10)	0.0395 (9)	0.0387 (9)	−0.0009 (8)	0.0034 (8)	−0.0049 (7)
C5	0.0504 (11)	0.0312 (9)	0.0507 (10)	−0.0010 (8)	−0.0027 (9)	−0.0027 (8)
C6	0.0481 (11)	0.0380 (9)	0.0470 (9)	0.0008 (8)	−0.0044 (8)	0.0016 (7)
C7	0.0478 (11)	0.0347 (9)	0.0430 (9)	−0.0017 (8)	0.0075 (8)	−0.0048 (7)
C8	0.0414 (10)	0.0367 (9)	0.0365 (9)	−0.0037 (8)	0.0053 (8)	−0.0042 (7)
C9	0.0444 (10)	0.0344 (9)	0.0433 (9)	−0.0038 (8)	0.0048 (8)	−0.0047 (7)
C10	0.0460 (11)	0.0468 (10)	0.0425 (9)	0.0009 (8)	0.0041 (8)	−0.0099 (8)
C11	0.0821 (15)	0.0456 (11)	0.0513 (11)	0.0044 (10)	−0.0003 (10)	−0.0010 (8)
C12	0.0490 (12)	0.0516 (11)	0.0507 (10)	0.0063 (9)	−0.0054 (9)	0.0003 (8)
C13	0.0603 (14)	0.0705 (14)	0.0663 (13)	0.0025 (11)	−0.0183 (11)	−0.0055 (11)

### Geometric parameters (Å, °)

**Table:**

O1—C9	1.287 (2)	C12—C13	1.498 (3)
O2—C9	1.248 (2)	C2—H2	0.9300
O3—C4	1.3595 (19)	C3—H3	0.9300
O3—C12	1.431 (2)	C5—H5	0.9300
O1—H1	1.01 (2)	C6—H6	0.9300
C1—C2	1.387 (2)	C7—H7	0.9300
C1—C6	1.400 (2)	C10—H10A	0.9700
C1—C7	1.464 (2)	C10—H10B	0.9700
C2—C3	1.379 (2)	C11—H11A	0.9600
C3—C4	1.382 (2)	C11—H11B	0.9600
C4—C5	1.382 (2)	C11—H11C	0.9600
C5—C6	1.370 (2)	C12—H12A	0.9700
C7—C8	1.337 (2)	C12—H12B	0.9700
C8—C9	1.479 (2)	C13—H13A	0.9600
C8—C10	1.504 (2)	C13—H13B	0.9600
C10—C11	1.511 (2)	C13—H13C	0.9600
			
C4—O3—C12	118.54 (12)	C1—C6—H6	119.00
C9—O1—H1	112.9 (10)	C5—C6—H6	119.00
C2—C1—C6	116.29 (14)	C1—C7—H7	115.00
C6—C1—C7	124.44 (14)	C8—C7—H7	115.00
C2—C1—C7	119.13 (14)	C8—C10—H10A	109.00
C1—C2—C3	122.74 (14)	C8—C10—H10B	109.00
C2—C3—C4	119.41 (15)	C11—C10—H10A	109.00
O3—C4—C5	115.64 (14)	C11—C10—H10B	109.00
C3—C4—C5	119.30 (15)	H10A—C10—H10B	108.00
O3—C4—C3	125.05 (14)	C10—C11—H11A	109.00
C4—C5—C6	120.54 (14)	C10—C11—H11B	109.00
C1—C6—C5	121.71 (15)	C10—C11—H11C	109.00
C1—C7—C8	130.35 (15)	H11A—C11—H11B	109.00
C7—C8—C9	118.18 (14)	H11A—C11—H11C	109.00
C9—C8—C10	115.44 (14)	H11B—C11—H11C	109.00
C7—C8—C10	126.37 (14)	O3—C12—H12A	110.00
O1—C9—O2	121.94 (14)	O3—C12—H12B	110.00
O1—C9—C8	118.35 (14)	C13—C12—H12A	110.00
O2—C9—C8	119.72 (14)	C13—C12—H12B	110.00
C8—C10—C11	114.16 (15)	H12A—C12—H12B	109.00
O3—C12—C13	107.38 (15)	C12—C13—H13A	109.00
C1—C2—H2	119.00	C12—C13—H13B	109.00
C3—C2—H2	119.00	C12—C13—H13C	109.00
C2—C3—H3	120.00	H13A—C13—H13B	110.00
C4—C3—H3	120.00	H13A—C13—H13C	109.00
C4—C5—H5	120.00	H13B—C13—H13C	109.00
C6—C5—H5	120.00		
			
C12—O3—C4—C3	2.0 (2)	O3—C4—C5—C6	179.67 (15)
C12—O3—C4—C5	−176.99 (14)	C3—C4—C5—C6	0.6 (3)
C4—O3—C12—C13	177.96 (14)	C4—C5—C6—C1	−0.5 (3)
C6—C1—C2—C3	1.2 (2)	C1—C7—C8—C9	174.19 (16)
C7—C1—C2—C3	177.04 (16)	C1—C7—C8—C10	−6.8 (3)
C2—C1—C6—C5	−0.4 (2)	C7—C8—C9—O1	3.1 (2)
C7—C1—C6—C5	−176.02 (16)	C7—C8—C9—O2	−176.51 (16)
C2—C1—C7—C8	156.09 (18)	C10—C8—C9—O1	−176.06 (14)
C6—C1—C7—C8	−28.4 (3)	C10—C8—C9—O2	4.4 (2)
C1—C2—C3—C4	−1.1 (3)	C7—C8—C10—C11	103.29 (19)
C2—C3—C4—O3	−178.82 (15)	C9—C8—C10—C11	−77.65 (19)
C2—C3—C4—C5	0.1 (2)		

### Hydrogen-bond geometry (Å, °)

**Table:**

*D*—H···*A*	*D*—H	H···*A*	*D*···*A*	*D*—H···*A*
O1—H1···O2^i^	1.01 (2)	1.61 (2)	2.6184 (15)	176.8 (15)
C7—H7···O1	0.93	2.30	2.7218 (19)	107
C12—H12B···CgA^ii^	0.96	2.82	3.6534 (19)	145

Symmetry codes: (i) −*x*+1, −*y*, −*z*−1; (ii) *x*, *y*, *z*+1.
